# Dutch Christian Faith Leaders Deliberating Human Germline Gene Editing: A Qualitative Study

**DOI:** 10.1007/s10943-026-02637-y

**Published:** 2026-03-29

**Authors:** Wendy P. Geuverink, Janneke T. Gitsels, Christy Betgem, Diewertje Houtman, Carla G. van El, Martina C. Cornel, Bert Jan Lietaert Peerbolte, Linda Martin

**Affiliations:** 1 Department of Human Genetics, Amsterdam UMC, Location Vrije Universiteit Amsterdam, Amsterdam, The Netherlands; 2 Amsterdam Reproduction and Development Research Institute, Amsterdam, The Netherlands; 3 Department of Midwifery Science, Amsterdam UMC, Location Vrije Universiteit Amsterdam, Amsterdam, The Netherlands; 4 Amsterdam Public Health Research Institute, Amsterdam, The Netherlands; 5 Midwifery Academy Amsterdam Groningen, InHolland, Amsterdam, The Netherlands; 6 Department of Primary and Long-Term Care, University of Groningen, University Medical Center Groningen, Groningen, The Netherlands; 7 Department of Youth Health, GGD Regio Utrecht, Utrecht, The Netherlands; 8 Training Institute for Physicians in Public Health and Youth Medicine, SBOH, Utrecht, The Netherlands; 9 Erasmus Medical Center, Department of Clinical Genetics, Rotterdam, The Netherlands; 10 School of Religion and Theology, Vrije Universiteit Amsterdam, Amsterdam, The Netherlands

**Keywords:** Human germline gene editing, Heritable human genome editing, Qualitative research, Faith leaders’ perspectives, Religious values

## Abstract

While human germline gene editing (HGGE) is not allowed, the World Health Organization and others called for public dialogue to clarify the values at stake. Some values may be rooted in religion, such as Christianity, and relevant to religious communities and wider society. In-depth interviews (*N* = 16) were conducted with Dutch Christian faith leaders in 2019 and 2024. Participants were asked about their views and underlying values, their possible approaches in pastoral counselling and their recommendations for future legislation regarding HGGE. Reflexive thematic analysis was used to analyze the interviews. The findings of this study firstly showed that participants’ views on the (un)acceptability of HGGE were underpinned by six values: human dignity; humility; alleviation of suffering; acceptance; compassion and justice. Secondly, regarding pastoral counselling, participants indicated that they would try to support the person(s) in need unconditionally regardless of their church teachings and/or their own values regarding HGGE. At the same time, their values would guide a thoughtful reflection on someone’s motivation behind using HGGE. Finally, regarding future legislation, participants call for great caution and for legislation against human enhancement. They worry about a slippery slope from individual ‘therapeutic’ cases to unwanted expansions. This study contributes to a better understanding of values that lead to views regarding HGGE, which is relevant for dialogues and policymaking.

**Supplementary Information:**

The online version contains supplementary material available at https://doi.org/10.1007/s10943-026-02637-y.

## Introduction

### Religious Voices in The Dialogue on Human Germline Gene Editing

Since the discovery in 2012 of the gene editing tool CRISPR-Cas9, developments in human genome editing technologies have been moving fast (FDA, 2023; Wong, 2023). For patients suffering from severe hereditary diseases, the prospects appear promising, while at the same time, there are many concerns about the potentially significant impact of this technology on individuals, families and society (Council of Europe, 1997; International Bioethics Committee, 2015). In particular, human germline gene editing (HGGE), which takes place in a gamete or embryo and potentially passes on changes to all subsequent generations, raises many questions (Almeida & Ranisch, 2022; Andorno et al., 2020; Coller, 2019; Jasanoff et al., 2015). These questions concern, besides the safety and effectiveness of the technology, for example, the fear of eugenics and the moral status of an embryo.

Currently, a significant majority of countries worldwide, including the Netherlands, prohibit HGGE if a pregnancy is induced (Baylis et al., 2020; Overheid.nl, 2021). Various authoritative (inter)national committees and bodies have repeatedly expressed the need for societal dialogue on HGGE to translate society’s needs and values into thoughtful regulations and policies (de Wert et al., 2018; Lander et al., 2019; National Academies of Sciences, 2017; National Academy of Medicine, 2020; Nuffield Council on Bioethics, 2018; World Health Organization, 2021b). According to the WHO, HGGE is also the subject of intense debate because it might raise ‘spiritual, religious or deeply personal issues’ related to human reproduction (World Health Organization, 2021a). These potentially disruptive technologies raise questions about what it means to be human and what values guide emerging technologies to promote health.

For many, religion is the “language of morality” and specifically engaging religious voices will enrich the public dialogue on HGGE (Cole-Turner, 2008; Kalidasan & Das, 2022; Sandel, 2007). Therefore, the current study aims to elicit the voices of Dutch Christian faith leaders regarding HGGE through an exploratory interview study.

### The Importance of Christian Faith Leaders’ Perspectives

Worldwide, the percentage of people who are religious is increasing, from 81% religious believers in 1970 to 87% in 2000 and 89% in 2022 (Zurlo et al., 2022). Most of this increase takes place in the Global South and can be attributed to Christians and Muslims, with Christianity being the largest religion (Center for Global Christianity, 2024). However, the Netherlands, like the rest of the Global North, is experiencing a reverse trend where the number of religious people is decreasing. Still, 30% of the Dutch report being Christian, of whom 17% are Roman Catholic and 13% are Protestant (CBS, 2024; Center for Global Christianity, 2024). Many of these believers worldwide will be influenced by their faith leaders’ views. In addition, congregants will also turn to their religious leaders for pastoral guidance, which might include disease- and reproduction-related issues stemming from emerging (bio)technologies (Dozier et al., 2020). Faith leaders are therefore important agents whose opinions and actions influence culture and existing values, and thus practice, alongside other agents such as doctors and healthcare providers (Adedini et al., 2018). As such, it is important to engage faith leaders in the societal dialogue on HGGE and to obtain a clear picture of their views and underlying values regarding the (un) acceptability of HGGE.

In 2024, it was reported that there are 2.6 billion Christians worldwide affiliated with several distinct branches (Center for Global Christianity, 2024). The three main branches are the Roman Catholic Church, Protestant churches, including the Evangelical/Pentecostal churches, and the Eastern Orthodox Church (Grove et al., 2022). The latter is not considered in this manuscript because this branch is too small in the context in which this study was created. The three branches mentioned recognize each other as part of the global Christian faith, despite their differences (Grove et al., 2022).

### Roman Catholic Perspectives on Human Germline Gene Editing

To address bioethical issues, such as human germline gene editing, the Roman Catholic Church released a document in 2008 by the Congregation for the Doctrine of the Faith, entitled ‘Dignitas Personae’ (The Dignity of a Person) (Vatican, 2008). At the heart of this document is the dignity of every human being to be recognized “from conception to natural death”.

With regard to germline editing for ‘therapeutic’ purposes, the risks to potential offspring are considered high given the current state of research and it is therefore not morally permissible. Moreover, germline gene editing requires in vitro fertilization (IVF), in which mature eggs are taken from ovaries and fertilized by sperm in a laboratory. However, for the Roman Catholic Church, it is only ethically acceptable if a child is conceived naturally through the loving union of a married couple. Furthermore, IVF often leads to the destruction of ‘leftover’ embryos. If HGGE were to be used for human enhancement, the concern is that it will promote a eugenic mentality and lead to discrimination against people who do not possess certain characteristics. Given these ethical objections, all applications of germline gene editing are morally impermissible to the Roman Catholic Church in the current situation. (Vatican, 2008).

### Protestant Perspectives on Human Germline Gene Editing

Unlike the Roman Catholic Church, within the Protestant Churches there is not one central authority and therefore no official church position on HGGE. Various statements, however, have been issued by large Protestant institutions in recent years.

In 2022 the Central Committee of The World Council of Churches representing over 580 million Christians worldwide, issued a statement on new and emerging technologies in which they “strongly support calls for a worldwide moratorium on applying CRISPR gene editing technology to the human genome”. While affirming that technology can contribute to healing, the concern is that HGGE could be used “for unethical or cosmetic purposes” (World Council of Churches, 2022).

In 2006 the National Council of the Churches of Christ, USA, which includes as members most U.S. Protestant denominations, issued a policy on human biotechnologies (National Council of Churches, 2006). It stated that “effective germline therapy could offer tremendous potential for eliminating genetic disease, but it would raise difficult distinctions about "normal" human conditions that could support discrimination against people with disabilities”. They called on researchers and healthcare practitioners to remain in ongoing dialogue with the general public and people from different religious backgrounds about these emerging technologies.

### Knowledge Gap and Study Aim

The official views and theological reflections of various Christian churches and scholars of religion regarding HGGE have been described before (Brauch, 2023; Cole-Turner, 2008). Likewise, religion as a demographic variable is regularly included in HGGE public engagement studies (Jedwab et al., 2020; Jibrilla et al., 2024; McCaughey et al., 2016; Thaldar et al., 2022). However, to our knowledge, no research has been conducted to explore in-depth the views and values regarding HGGE of Christian faith leaders holding leadership and pastoral positions (Geuverink et al., 2024b). By focusing on gathering participants’ values, we align with previous research in which a thorough understanding of underlying values related to HGGE is considered crucial for policy-making (Geuverink et al., 2024b; Nelson et al., 2021; Sarewitz, 2015; World Health Organization, 2021a).

In this paper, we adhere to the following definition of ‘values’: "beliefs, individual or social, about what is important in life and thus about the goals or objectives that should determine and shape public policy” (Royal Commission on Environmental Pollution, 1998). In this exploratory interview study, we decided to exclusively include Christian faith leaders from a wide range of Christian denominations because in the Global North Christians are the largest group of believers and Christian values are culturally deeply rooted.

The aim of this study is to provide in-depth insights into the views and underlying values of Christian faith leaders of various denominations in the Netherlands regarding human germline gene editing by answering the following main question and related subquestions: I) What views and underlying values are voiced by Christian faith leaders when considering the (un)acceptability of HGGE? a) If HGGE were implemented in the future, how would Christian faith leaders approach this in pastoral counselling? b) According to Christian faith leaders what should be included in potential future legislation regarding HGGE?

## Method and Materials

### Methods

Semi-structured interviews were performed with 16 faith leaders of Christian denominations in the Netherlands between May and July 2019 and between August and December 2024. After the first part of the face-to-face interviews in 2019, the intention was to continue the second part of the face-to-face interviews in the following year, but this was delayed due to the COVID pandemic. Through performing qualitative interviews, the aim was to gain in-depth insights into the participants’ views and values on human germline gene editing.

Little research has yet been published on how values can be accurately collected, although their importance is underlined. Participants usually articulate concerns, fears, needs and desires when it comes to what they consider of high value. In this study, the research team collectively sought ways to ‘translate’ participants’ reflections into concrete values.

A faith leader of a Christian denomination was defined in this study as someone who holds or held a leadership position in a church or congregation and offers pastoral counselling. An interview guide was constructed based on the literature published by the Beginning of Life Group hosted by the Vrije Universiteit Amsterdam (Goekoop et al., 2020; Smalbrugge & Cornel, 2020; Van Dijke et al., 2018, 2021) consisting of questions about their church/congregation; faith/religious beliefs; views on human germline gene editing; beginning of life; moral status of an embryo; genetic identity; pastoral care; and expectations of the future (Appendix 1: Interview guide). The Beginning of Life Group started in 2017 as a faculty overarching multidisciplinary research project focusing on emerging genetic technologies.

### Recruitment

A wide diversity of Christian denominations can be found in the Netherlands. For this study, participants were recruited using purposive sampling, as the aim was to select a heterogeneous group of faith leaders from a broad spectrum of Christian denominations (from liberal to moderate to conservative (Smith, 1990) without claiming completeness in this respect. Participants were recruited through the network of five members of the research team and extended from there through snowballing. Recruitment continued until data saturation was reached.

The prospective participants first received an email with information about the study (Supplementary Information: Information Letter). They were asked to contact the research team if they were interested in participating in the study. After agreeing to participate, study participants received an email with a link to a video to provide them all with the same basic information about CRISPR-Cas9 prior to being interviewed (SchoolTV, 1996).

All study participants were given verbal and written information about the purpose of the study prior to the interview. They were informed about confidentiality, that quotes would not be traceable to the individual and the option to withdraw at any time without giving a reason. After obtaining written consent from the participants the interviews were recorded and transcribed verbatim. The information from the participants was pseudonymized in the transcripts in order to protect their privacy. Participants were informed that interview transcripts would be kept for 10 years after the last publication and that audio recordings would be destroyed after they were transcribed.

### Analysis

After transcription the interviews were analyzed using MAXQDA software (version 2024). Reflexive thematic analysis (Braun & Clarke, 2006, 2019, 2021) was used for coding and analyzing the transcripts as described: (1) becoming familiar with the data, (2) generating initial codes, (3) searching for categories, (4) reviewing the themes, (5) defining and naming the themes, and (6) writing the report. The analysis was mainly conducted inductively, with the dataset being the starting point for engaging with meaning, rather than based on a predetermined conceptual framework (Braun & Clarke, 2021). The themes were selected, through discussion within the research team, according to their prevalence and/or their apparent importance in connection with the research question. The quotes included here were translated (by W.G.) from Dutch to English in ChatGPT 4o using an AI prompt and reviewed for accuracy by the research team (Maastricht University, n.d.).

### Results

A total of forty-two Christian faith leaders were invited to participate from which a total of sixteen agreed to participate. Fourteen faith leaders did not respond to the invitation e-mail. Reasons given by the (twelve) others for not participating were mostly limited time, or no further explanation was given.

Most participants (94%) had completed a university master’s degree or above, which can be explained by the fact that in most denominations a master’s degree in theology is required to become a minister or a priest. All participants were Dutch, living in various parts of the Netherlands. The participants were asked to self-identify their denomination as liberal, ‘middle of the road’ referred to as ‘moderate’ in this paper, or conservative and explain their choice (Appendix 2: Self-placement of Denomination). Participant characteristics can be found in Table 1.

**Table 1:** Characteristics of participants, N = 16

Characteristics	Participants n
*Gender of participants*	
Male	12
Female	4
*Age of participants (mean age 57)*	
31–35	1
36–40	2
51–55	2
56–60	4
61–65	5
71–75	1
81–85	1
*Denomination*	
Conservative Protestant	7*
Moderate Protestant	2
Liberal Protestant	3
Conservative (Roman) Catholic	1
Moderate (Roman) Catholic	2
Moderate (Old) Catholic	1
Level of Education (ISCED**)	
Bachelor or equivalent	1
Master or equivalent	13
Doctoral or equivalent	2

*Two participants in this category classified their denomination as conservative based on positions related to medical ethical issues while on other topics, they considered their denomination to be more moderate or liberal, **ISCED: International classification of education

The interviews were in Dutch and conducted by two authors (W.G. or C.B.). Interviews were conducted face-to-face, apart from one which was conducted over the phone. The interviews lasted between 37 and 99 min with an average of 67 min. Only the interviewer and participant were present during the interviews. The interview questions were not provided in advance.

Almost all interviews were characterized by extensive reflections and deliberations by the participants when it came to their views and underlying values regarding HGGE. Of the 16 participants, nine said they were in favor of using HGGE for ‘therapeutic’ purposes (P1, P4, P5, P7, P12, P13, P14, P15, P16) and seven were against (P2, P3, P6, P8, P9, P10, P11).

Both participants who indicated being in favor and against HGGE belonged to a wide range of denominations, from conservative to moderate to liberal. Several participants indicated that for them there were many different aspects to HGGE. This applied to both the participants who were against and in favor of HGGE. Below, the answers to the research question (I) and subquestions (a,b) are presented based on the results found.

### I) Views and Underlying Values of Christian Faith Leaders Regarding HGGE

The views and underlying values of Christian faith leaders regarding HGGE relate to three themes constructed by analyzing the interview transcripts: 1) Humans created in God’s image 2) The implications of HGGE for individuals and prospective couples, and 3) The societal consequences of the use of HGGE. All themes contain associated values (Table 2).

**Table 2:** Themes and values

Themes	Values
Humans created in God’s image	• Human dignity • Humility
The implications of HGGE for individuals and prospective couples	• Alleviating suffering • Acceptance (of life as it is)
The societal consequences of the use of HGGE	• Compassion • Justice

### Theme 1: Humans created in God’s image

#### Human dignity

A central theme is a fundamental Judeo-Christian conviction regarding human nature, in which humans are created in the ‘image of God’. A number of participants, mostly but not only, opponents of HGGE, referred to this theological doctrine of *imago Dei*. Participants considered this religious conviction the basis for the value of unconditional worth and dignity of all human beings, all made by God and considered ‘good’ regardless of their talents or genetic make-up. Some feared that this value could potentially be at stake using HGGE.

Several participants rooted this value on a Bible text from Genesis: “Then God said, “Let us make humans in our image, according to our likeness.” (Gen 1:26) (New Revised Standard Version-updated edition). Another text that served to emphasize human dignity was Psalm 139:13 (NRSVue), which reads: “For it was you who formed my inward parts; you knit me together in my mother’s womb.”, and further on in verse 16: “Your eyes beheld my unformed substance.” For some, these texts indicate that every human being is seen and known by God from the beginning.

*“You knew me already when I was in the womb, Psalm 139 (…) when I think of our daughters, I only got to know them for the first time at birth, so when they were out of the womb… but God already knew them before that time.” (#2, conservative, Protestant)*

The destruction of ‘leftover’ embryos was considered highly undesirable by some participants based on this theological view of human beings. Whether a participant considered an embryo a (potential) human being, or worthy of protection from conception onwards, played a role in deciding to be for or against the use of HGGE, but it was not decisive. Most participants against HGGE considered an embryo highly worthy of protection, but sometimes other values (i.e., acceptance of life as it is) were mentioned as decisive for being against HGGE.

One participant said that because of their belief in reincarnation and the related specific earthly assignment for each embryo, they were against HGGE which would disrupt this assignment. Two other participants indicated that they were not categorically opposed to the modification of embryonic DNA to prevent genetic conditions. However, they saw unacceptable obstacles in the way this modification is achieved, namely by creating an embryo outside the mother’s body through IVF, or in the destruction of the leftover embryos. For them, HGGE would become (more) acceptable if it were possible to avoid embryo loss or the use of IVF.

*“If the embryo is in the mother’s body, maybe there could still be a possibility [to modify DNA]. But if the embryo is outside the mother and then reimplanted... that makes it very problematic... The primary purpose [of sexual intercourse] is an expression of total love, and through that, a human life can come into being. If it’s shifted to the medical world, then self-giving is lost and love... that becomes questionable. God intended for a child to be conceived through the union of two bodies. That is how it should remain.” (#8, conservative, Roman Catholic)*

When it comes to creating embryos for research purposes, which is necessary to research the safety and effectiveness of HGGE some expressed support in order to “improve the safety of the technique” or saw the use of embryos as “collateral damage”. Others added that it should be allowed to a very limited extent and only under strict conditions. Some were against creating embryos for research or conducting experiments on embryos because of an intuitive sense of repulsion or for them embryos were of great value and should not be treated as a “thing” or a “guinea pig”.

*“Well, for example, experiments on embryos. So far, it’s still prohibited—but maybe that’s exactly what’s needed for such a promising development, and so on. Still, it worries me… the ethics of it, how it shifts little by little—‘just this little thing, then it’s fine’—but really, it’s like the ‘very hungry caterpillar that’s never satisfied’. Science always pushes forward with something new.” (#3, conservative, Protestant)*

#### Humility

Many participants mentioned their concerns about the human desire to control life through the limitless use of technology. Some considered this a form of pride and contrary to the value of humility. Several reflected on the place or role of human beings in creation when it comes to the use of HGGE. The role of stewardship is variously interpreted in this context. Participants who opposed HGGE, more often referred to people’s desire to “play God”, to “want to sit on God’s throne” or to “want to be like God”. A few participants mentioned the ‘rebellion’ of Adam and Eve in paradise as an example of where the desire ‘to be like God’ can lead to.

Among some participants in favor of HGGE, there was more emphasis on the fact that God has given people intellect and medical knowledge for them to use well, or on the fact that the Bible does not give clear guidelines regarding HGGE.

*“Well, yes, that ‘Babel culture’, the great Babel we’re going to build with technology, so to speak. And to control everything and then gain power over it. Well, I think that’s quite a striking metaphor (…) And there’s pride in that. Arrogance. Human arrogance. Human overestimation of oneself.” (#10, conservative, Protestant)*

*“I think God gave humans intelligence and not without reason. We’re meant to use it, but also to be very aware of what we do, and why we do it. Take that man in China who made babies AIDS-free and then I think: yes, that’s going way too far, that’s not something we should want. He broke all kinds of scientific rules.” (#5, conservative, Protestant)*

A few participants said they feared people might decide to use HGGE based on egocentric motives. They indicated that these egocentric motives come from an overly individualistic view of life, in which it is mainly about one’s own desires and not also about the value of caring for “the other”, for “nature” or without awareness of “God” or “the secret of life”.

*“Absolutely wonderful, and if by fiddling a little with heredity you can prevent a great deal of suffering, well, that’s fantastic that it’s possible. So, I’d be very much in favor of that. The only question, of course, is: to what end? That’s what it comes down to, I think. If it’s only with a focus on myself—I want a good life, I want a perfect family, I want cool kids—then it gets problematic. But if it’s about ‘life’ and ‘living together’ and about ‘the mystery of life,’ then it’s fine.” (#14, liberal, Protestant)*

### Theme 2: The implications of HGGE for individuals and prospective couples

#### Alleviating suffering

The main reason for being in favor of HGGE was the possibility of ‘preventing’ serious diseases and thereby connects to the value of alleviating suffering for individuals and prospective couples. Some participants referred to texts or a central theme they saw in the Bible such as “loving your neighbor as yourself” or “do to others what you would have them do to you”, as the foundation of this value. A few participants were in favor of HGGE without reluctance while others hesitated and made explicit value trade-offs, for example regarding societal values that might be at stake if HGGE were allowed on a broader scale.

One participant was in favor of removing all medical conditions through HGGE, without distinguishing between severe and mild conditions The other eight participants explicitly said that they were in favor of using HGGE only for serious genetic conditions. Whereby several participants also indicated that the distinction between mild and serious conditions is very difficult to make.

*“I think there are so many sides to this [human germline gene editing] that it’s really difficult to simply say it’s good or it’s bad. In our own congregation, for example, we have people who are dealing with Huntington’s disease (…). That’s a very clear example of an extremely serious illness that might be resolved through this. And not in any other way. That puts you in the middle of a very complex ethical dilemma. If you look purely at the individuals then you say yes you know, you would want that for these people. If it can help, what are we bothered about? Whereas if you try to zoom out and take a broader view, all sorts of questions arise (...) those diseases that are being mentioned, who’s to say it will only be used for those? (…) Both in terms of what will be created, what might come into existence, and how will ordinary humanity respond to it? What is even ‘normal’ anymore? There’s also the thought of creating superhumans. I find that very concerning. Really tampering with what the Creator has given.” (#13, conservative, Protestant)*

*“If it’s simple and it’s one gene that determines whether you get the disease, and it doesn’t affect anything else, and you have a list of 100 such diseases, from severe to less severe… then I say: ‘while you're at it [human germline gene editing], work through that whole list right away.’” (#4, moderate, Protestant)*

Many participants mentioned (medical) safety as a crucial prerequisite for HGGE, pointing in particular to the risk of unintended consequences, which can occur immediately or many years after editing an embryo.

*"You’re tinkering with the deepest layer of the human being, with human genetics. But do you oversee everything you’re doing in that? I wonder about that. That’s what makes these things difficult, when 25, 30, or 40 years later you say: ‘We did overlook something after all.’ That’s where my hesitation lies. That’s why I’m also quite reserved about it." (#7, moderate, Protestant)*

#### Acceptance (of life as it is)

Although many participants mentioned the value of alleviating suffering for individuals and prospective couples through the use of HGGE as something positive, this was not a decisive value for everyone. Several participants indicated that although they recognized that HGGE would lead to alleviating suffering in terms of preventing serious hereditary conditions, in other ways, using this technique could actually cause more suffering.

Participants mentioned, for example, the increase in available choices for future parents regarding their offspring because of these emerging technologies, and the increasing personal responsibility for the consequences of their choices. For example, how will future children react to the choices parents made for them when they were not yet conceived? Or how will people around you react if you choose ‘nothing’ and end up having a child with an hereditary condition that could have been ‘prevented’? Based on these aforementioned concerns, some participants were not in favor of using HGGE.

*“You also inflict suffering on the prospective parents, by the way. That’s when you realize the moral impact of what you’re doing, that it affects the people who are asking for it themselves, but who may not yet be aware of that. They are making a decision for something that doesn’t yet exist. That something may later A. have its own opinion about it. B, it means that something that is theirs, is not allowed to exist. So, it also says something about—it’s also a judgment about yourself. That causes a lot of harm to people. Because these are often people who are already struggling in life; otherwise, they wouldn’t be at the clinical geneticist. There’s already something going on. So, at its core, it basically says: I, or you, or we together, are not allowed to exist this way. This is not right.” (#9, moderate, Old Catholic)*

A number of participants also mentioned the importance of letting nature and natural processes take their course. Reference was also made to a certain normative order of creation in which only God is the creator and in which humans have been given the task of stewardship. Some pointed in this context to climate problems caused by human intervention.

Furthermore, many participants saw a similarity between questions that play a role at the beginning of life (HGGE) and at the end of life. Simply because certain medical technologies are available does not mean using them will always add to quality of life. Several participants said they felt it was important to think carefully about the purpose of any medical treatment and who would benefit from it. They provided examples of what they saw as needlessly prolonging the lives of old people who are seriously ill or, on the contrary, hastily requesting euthanasia out of fear of the final stage of life or because of the thought of being a burden to others. In both the reflections around HGGE and these ‘end-of-life situations’, participants saw a contemporary struggle to endure suffering, slow down and relate to what life brings.

*“You sometimes see it with euthanasia as well. The way people approach euthanasia. I once had a very wealthy couple [in the congregation]. Extremely rich people, and the woman had cancer. Well, of course, that was very tough. But she was someone who went to every hospital, even all the way to the U.S., and could have the most expensive treatments. And when that didn’t help, she immediately wanted euthanasia. And then you think: you’re completely skipping the question: ‘What does this mean for my life?’, ‘What do I want to do with my life?’, ‘How do I want to end my life?’” (#11, liberal, Protestant)*

### Theme 3: The societal consequences of the use of HGGE

Several participants mentioned knowing people, often also from their congregation, who suffered greatly from a hereditary condition in their family. For this reason, some wanted to allow the use of HGGE for these individuals and couples. At the same time, many participants expressed considerable concerns about the potential negative impact of HGGE on society should it be more widely used.

#### Compassion

Some participants expressed their concerns that in a society primarily focused on eliminating suffering and increasing self-determination due to growing technological possibilities, the value of compassion might be at stake. Caring for one another, for the weak or the sick, and also for each other as a community, can evoke something good in people and give meaning to people’s lives. Several participants here referred to a central message they found in the Bible where the most vulnerable are given a place of honor. Questions were raised, such as do we still dare to trust that we will be taken care of when we are demented or old or have a child who needs extra care?

A few participants added that this potential loss of compassion is not a good argument for not addressing severe genetic conditions, but at the same time stressed that questions about what the use of these technologies might mean for society in this regard should be asked.

*I think that [someone with an illness or disability] evokes something in every person. How can I be of support? How can I contribute to someone’s life? How lucky I have actually been that my life isn’t like that. And if those people are no longer there, that appeal to our compassion is no longer made. Of course, you can’t simply turn that reasoning around and say: that’s why we like it when there are still a few people with dementia around us, because then… That’s not possible. But that doesn’t take away from that first thought. That as we become increasingly successful at eliminating deviation, illness, and cognitive decline from our society, we also become more afraid of it. Because then we simply no longer encounter it in our surroundings. (#16, liberal, Protestant)*

#### Justice

A few participants said HGGE would compromise the value of justice and could lead to more inequality because only people with money could use it. Furthermore, some feared that patients in countries with fewer resources would not have access to this technology.

*“So, I think, yes, we might be able to solve it once, but then here in the wealthy West we have the method to do so, while where it’s really needed, on the other side of the world, where there is immense suffering, we solve nothing. Because they won’t have the money or the means to do this. Okay, what gives us the right to exploit a very small part of the world like this. That’s yet another layer. Ultimately, the answer to how we should relate to the world is through equal distribution. Well, this [human germline gene editing] doesn’t lead to that. I think it only creates more inequality. So that’s another layer. The more you zoom out, the more you look at it on a macro level, the more unfair I think it becomes.” (#9, moderate, old catholic)*

Furthermore, many participants said they feared a new form of eugenics if HGGE would be allowed. Several referred to the eugenic practices of the Nazis during the Second World War, such as the forced sterilization of groups of people they considered ‘inferior’ in order to create genetically ‘superior’ people. By using HGGE, people with certain conditions, such as Down syndrome, or specific traits, such as ADHD, might no longer be welcome. This could also lead to limited access to resources or reduced care for people with disabilities and an increase in discrimination against them.

All participants indicated that they are against human enhancement, which is, in their opinion, also aimed at creating the ‘ideal’ human being. The question of who gets to determine what is ‘ideal’ came up often in this context and goes against the values of justice and human dignity in which every human is treated as equally valuable and has dignity regardless of certain traits. Some indicated that the line between therapy and enhancement is sometimes difficult to draw. Finally, a few participants added that from a theological perspective, people can improve biotechnologically, but in a spiritual sense they do not become better in ‘the evil they can cause’.

*“Many years ago, there lived a truly wicked man, his name was Adolf Hitler. His goal was to create an Aryan Übermensch. When I think about embryo modification, I get that same kind of unsettling feeling in my stomach. If things start heading in that direction… then… oof. Then I’m strongly opposed. When it comes to the idea of manufacturability, let’s put together a baby… would you want that? No. It is God who said: “Let us make humans in our image, in our likeness.” In all their diversity, people are complementary to each other.” (#1, conservative, Protestant)*

A few participants questioned whether HGGE could be considered as therapeutic at all, or whether it was rather aimed at preventing the birth of people with certain characteristics, which compromises the closely connected values of justice and human dignity.

*That [human germline gene editing]is not entirely therapeutic; it’s about influencing life. It’s about steering and directing a society. That’s where the real issue lies for me. Then medicine itself takes on a completely different meaning and a completely different function: solving the problems of our society. You can start changing people. That’s a completely different way of thinking than what medicine is about. Medicine has always been reactive: we discover a problem, and we develop something in response. And now it becomes: ‘We don’t want these people, and they will no longer be born.’” (#6, conservative, Protestant)*

### a) Pastoral counselling regarding HGGE

If HGGE would be implemented in the future to ‘prevent’ serious genetic disorders, religious leaders will most likely receive pastoral questions about this technology as well. Twelve of the 16 faith leaders explicitly mentioned that in the pastoral context, the value of inclusive pastoral care, where they try to provide (unconditional) support to a person or couple in need, is more important than their own, sometimes fundamental, objections. A number of participants added that they would ask questions to elicit someone’s motives or objectives.

Some participants referred to Jesus’ teachings in which humans take precedence over rules. For example, when Jesus heals someone on the Sabbath, which was prohibited, the person’s suffering outweighed the rules for him (Luke 14:1–6). Participants provided examples of current pastoral questions related to preimplantation genetic testing (PGT), euthanasia or abortion in which the person in need took precedence over doctrines of the church or their own values and beliefs. Several participants added that sacraments such as anointing the sick prior to euthanasia, leading a Catholic funeral service after someone chose euthanasia or baptizing a child born after PGT, would always be carried out by them, if requested.

*“But ‘my own church’, you also need to nuance that. Because of course you have the official doctrinal positions, and then you have the people who have to deal with them. And fortunately, I see many colleagues, and even occasionally bishops, who approach this in a very pastoral way and who also say: in the end, this is a decision for those involved themselves.” (#15, moderate, Roman Catholic)*

*“If Christ brought us anything, it is surely a profoundly liberating theology that says: all those rules may be fine, but they are ultimately secondary, what matters most is the human being.” (#9, moderate, Old Catholic)*

### b) Recommendations for legislation regarding HGGE

When it comes to future legislation around HGGE, a number of participants expressed the need for great prudence as they worried about a slippery slope in case HGGE were allowed for severe hereditary conditions. Some referred to the expansion of legislation for both abortion and euthanasia in the Netherlands over the years as an explanation for these concerns. These developments gave participants little confidence that the legislation would provide adequate protection regarding the use of HGGE. Nevertheless, they felt that clear legislation regarding HGGE is needed.

*“I think I would be in favor of allowing it in cases of serious illness. But my concern would be that… analogous to euthanasia practice... that the boundaries just keep getting pushed further and further. […] There’s a parallel there too with the practice of euthanasia. In 2002 when the [Dutch euthanasia] law came into effect, let’s say, there was a generally accepted idea that once dementia had been diagnosed, it was simply impossible [to get euthanasia]. Someone was no longer considered mentally competent. One can see where we are now more than 20 years later. And legislation is also in the hands of politicians. So, we see there too how suddenly, if the political constellation changes, everything can change again. So, in that regard, yes. I don’t have much confidence in it.” (#16, liberal, Protestant)*

Some participants mentioned the need for legislation against coercion by government or insurance companies. Moreover, some participants said they were concerned that this technology could fall into the hands of commercial parties.

*“That would mean that an insurance company, if it were to gain access to that information, could say: “Then we don’t want to insure you, because the chances of you getting sick are serious. We don’t know whether you’re a carrier of the same gene your mother or father carries, but the fact that you have not tested for carrier status makes you a high-risk patient for us. Therefore, we can only insure you at a higher premium.” I personally find that inhumane. Because, even more fundamentally, that would mean that we are moving in that direction, the ‘desired human being,’ the ‘perfect human being’. Just a few more steps and that’s where we’ll end up. You set a standard for what a human should be or is allowed to become. I would not approve of that myself.” (#6, conservative, Protestant)*

Many participants mentioned the need for prohibition against human enhancement in the law. If HGGE would be allowed at all, then only to ‘prevent’ serious hereditary conditions and not to change appearance (e.g. eye color), to make people faster, stronger or smarter or determine the sex of the future child.

*“Well, at least some kind of safeguard [in the law] against the misuse [of HGGE]. So that it’s clearly for the healing process and not for an enhancement process. In the sense of making things faster and that sort of thing. So, I think, no, it really should be about making currently incurable diseases curable.” (#12, moderate, Roman Catholic)*

## Discussion

This qualitative study aimed to explore the views and underlying values of Dutch Christian faith leaders regarding HGGE, the consequences for their pastoral counselling if HGGE were implemented, and their recommendations for legislation. The findings of this study firstly suggest that participants' views on the (un)acceptability of HGGE were underpinned by six values, namely: human dignity; humility; alleviation of suffering; acceptance; compassion and justice. Secondly, when it comes to pastoral counselling regarding HGGE for ‘therapeutic’ purposes, participants indicated that they would try to support the person(s) in need unconditionally, regardless of their church teachings or their own values. At the same time, their values would guide a thoughtful exploration of someone’s motivation behind the use of HGGE. Finally, when it comes to future legislation, the participants call for great caution and legislation against human enhancement. They are concerned about a slippery slope where legal permission for individual ‘therapeutic’ cases could ultimately lead to undesirable expansions.

We found that participants from more liberal denominations are not necessarily more positive about HGGE than those from more conservative denominations. Participants from across the broad spectrum of churches, from conservative to moderate to liberal, and within both the (Roman) Catholic and Protestant churches, show similar variations in their value trade-offs.

The values in this study are expressed within a clear religious framework, with its own moral vocabulary and underpinnings shedding new light on why certain values are considered desirable or at stake, and how they provide direction to participants’ views on HGGE.

As an example, in this study the value of human dignity is being explicitly informed by the theological view of human nature. In this view, all human beings are created in God’s image and therefore have inherent dignity, regardless of their abilities, traits or physical condition. Regarding the handling of embryos, we found that the intrinsic value of an embryo, often guided by the value of human dignity, was a reason for several participants to oppose HGGE.

However, this opposition was not always, or not entirely based on a fundamental rejection of modifying embryonic DNA. For example, one Roman Catholic and one Protestant participant explicitly added that they do have a positive attitude towards HGGE when it is used for ‘therapeutic’ reasons to alleviate suffering, but that they are against the way it is performed, based on the value of human dignity. This view is in line with the official Roman Catholic viewpoint which rejects HGGE for medical purposes merely because of ethical objections to the procedures, such as IVF, the risk of unintended errors, and the deliberate destruction of embryos (Vatican, 2004, 2008).

The International Theological Commission, an advisory body of the Vatican, mentioned as a possible alternative: “the use of gene therapy in the stem cells that produce a man’s sperm, whereby he can beget healthy offspring with his own seed by means of the conjugal act” (Vatican, 2004). In recent studies, new in vivo methods for genome editing are presented, such as i-GONAD (Genome-editing via Oviductal Nucleic Acids Delivery) and TIGER (Targeted In vivo Genome Editing in Rodents). In these studies, which are currently being carried out exclusively on rodents, the ex vivo treatment of embryos is no longer necessary because the CRISPR-Cas9 components are directly delivered into the oviduct of the pregnant female (Li et al., 2023; Sato et al., 2023). The use of these new technologies is far from feasible for human beings, but in theory, it could remove the procedural objections to HGGE, which could influence the acceptability of HGGE for certain Catholic believers and others as well.

A second example from this study of how values from a religious framework may inform views on HGGE can be seen in the value of humility. Here the role of humans in creation versus that of God as creator comes into play. On the one hand, there are concerns that the use of HGGE could be guided by human arrogance or selfishness in which humans overstep their given place. On the other hand, God-given technology can also be well used to alleviate human suffering. Theological literature also explores this theme. In this literature, the idea of ‘playing God’ is balanced against the idea of humans as ‘created co-creators’ who use technology for good (Cole-Turner, 2002, 2008).

A third example is how the values of justice and human dignity play a role in the rejection of human enhancement, in which certain characteristics are considered more valuable than others. Participants are concerned that HGGE could be used as a eugenic tool in which human judgements are at the root of a person’s existence. Participants explicit rejection of human enhancement is in line with the official Roman-Catholic position and the statements of the World Council of Churches (2022) and the National Council of Churches (2006) and with previous interview studies with religious stakeholders who were explicitly against human enhancement (Geuverink et al., 2023, 2024a). However, it contrasts with some general public studies, where around 30% of the participants were in favor of human enhancement, particularly improving intelligence (Jedwab et al., 2020; McCaughey et al., 2016; Scheufele et al., 2017).

As these studies did not search for values, it is unclear based on which values these participants were in favor of enhancement. When it comes to HGGE to ‘prevent’ severe genetic conditions, we found in our study that slightly more than half of the participants could support this application based on the value of alleviation of suffering. This is more in line with the general public’s views in various large (global) engagement studies which often show a percentage of around 60% in favor of using HGGE for severe conditions (Hendriks et al., 2018; Houtman et al., 2022; Jedwab et al., 2020; McCaughey et al., 2016; Scheufele et al., 2017). Likewise, it is unclear from these surveys which values underline the participants’ views.

In the present study, as in the literature, we saw that faith leaders often provide pastoral counselling to members of their congregation on a variety of topics (Modell et al., 2014). Participants in this study explicitly mentioned that they try to provide unconditional support to a person or couple in need because they consider people more important than church doctrines or their own objectives. At the same time, participants appealed for reflection on the motives that play a role in deciding on HGGE, both on an individual level during counselling, and on a societal level. These motives, such as selfishness, or seeking to take ultimate control of life, conflict with the values participants hold dear. Cole-Turner’s pastoral reflection on biotechnology highlights the role that faith leaders can play in critically reflecting on the motives driving biotechnological advancements and considering them in the light of Christian values (Cole-Turner, 2002).

The public dialogue on HGGE and dialogues with medical experts (such as embryologists, reproductive physicians and clinical geneticists) can be enriched by the values from this study. Within the Dutch project ‘The DNA dialogues’, these retrieved values will be used to inform ‘professionals’ through (scientific) publications and by drawing on them in dialogues with experts from the knowledge chain and policymakers (Geuverink et al., 2024c).

### Strengths and limitations

Previous studies on HGGE have included religion as a demographic variable, but to our knowledge, religious faith leaders have not previously been interviewed about their views and underlying values regarding HGGE, where we could show a broad palette of values. The external validity of the results is limited and specific to the Dutch context. Due to the recruitment through the research team there is a high possibility of a selection bias which may have limited the diversity of perspectives and values in this study. Moreover, Christian faith leaders in the Netherlands may experience more individual freedom to deviate from official church teachings than Christian faith leaders in other less secularized countries. However, information power of our study is strong because it involves a group of participants with specific characteristics whose views, values and experiences have not been described before (Malterud et al., 2016). Moreover, no new themes came up in the last two interviews, which we considered a confirmation of sufficient sample size. Research involving faith leaders in other countries and parts of the world, both within Christianity and other religions, is important and a recommendation for future research.

## Conclusion

This study suggests six values are underlying Dutch faith leaders’ views on HGGE: human dignity; humility; alleviation of suffering; acceptance; compassion and justice. These values appear within a religious framework, with its own moral vocabulary and underpinnings, shedding new light on why certain values are considered desirable or at stake, and how they can provide direction when deliberating the (un)acceptability of HGGE.

HGGE proves to be a topic that evokes extensive reflections and considerations among the participants. Considerable diversity is found within and between different Christian denominations regarding the (un)acceptability of HGGE for individual ‘therapeutic’ purposes. At the same time, participants indicate that they would unconditionally support the person(s) in need in their pastoral counselling.

When it comes to the potential consequences for society, participants have many shared concerns about the impact of increasingly rapid advances in (bio)technology along with a perceived trend in society in which life is seen as ‘makeable’, focused on quick fixes and self-determination. Participants have limited confidence in legislation preventing a slippery slope from individual ‘therapeutic’ cases to unwanted expansions. They call for caution and for legislation against human enhancement.

This study provides a wider picture and better understanding of values that lead to views regarding HGGE, which can inform the dialogue on HGGE with the public and with (medical) experts and help policymakers inform their decisions.

## Electronic supplementary material

Below is the link to the electronic supplementary material.

Supplementary file1 (DOCX 46 kb)

## Data Availability

Data underlying this paper cannot be shared publicly because of participant privacy. The data can be shared on reasonable request to the corresponding author, for researchers who meet the criteria for access to confidential data.

## Interview guide

Title: Deliberations of Christian faith leaders regarding Human Germline Gene Editing.

Participants: Christian faith leaders of various denominations who have an active leadership role within a religious community, such as pastor, priest, minister or pastoral counselor.

### Interviewer's supplies:

- Audio recorder
- Consent form
- Pen
- Paper

### Introduction to the study

Dear < name > ,

Thank you for agreeing to participate in our study. My name is Wendy Geuverink. I am a PhD candidate at the department of Human Genetics at Amsterdam UMC and have a background in psychology and theology. I am a researcher involved in a national project called ‘The DNA Dialogues’. The aim of this project is to examine the views, needs and values in society surrounding Human Germline Gene Editing (the modification of the DNA of embryos with a genetic disorder) and how these can be translated into appropriate legislation and regulations. This interview study, in which we would like to hear the views of Christian faith leaders from different denominations is also part of that project.

Did you manage to watch the video on genetic modification prior to the interview? (https://schooltv.nl/video/de-kennis-van-nu-in-de-klas-gentechnologie/). If not, we will watch it together just now. Do you have any questions about it?

Before we proceed, I would like to go through a few practical matters with you that are also related to the consent form:

- The interview will last about 60 min.
- Everything you tell us will be kept confidential. If you prefer not to answer one or more questions, you do not have to.
- The interview is processed into a research report, in which your personal information cannot be traced. Only our research team will have access to the data. What you say will not be shared with anyone.
- There are no right or wrong answers, of course. We are simply interested in hearing your personal story, your opinion and ideas about modifying embryo DNA and your view on life.
- We make an audio recording of the conversation. The recording will be destroyed after we transcribe our conversation. If you wish, I can send you a copy of the transcript of our conversation.
- Do you have any questions for me at this point? I would like to reiterate that you may still withdraw from the study at any time, including during the interview.
- By signing the consent form, you indicate that you have understood everything and want to participate in our study.

Signing the consent form.

### Interview guide

In the interview that will start shortly, a number of topics will be addressed:

- Your (role in your) congregation/parish
- Worldview/faith
- Human Germline Gene Editing
- Dealing with embryos/beginning of life
- (Genetic) identity
- Pastoral care around this topic
- Vision of the future

At the end of the interview, I would like to give you a short questionnaire with some background information such as your age, education, your religious background, etc.

Do you have any questions?

Shall we start the interview? Then I’ll turn on the audio recording now.

### Start recording

Start by saying: ‘Start interview respondent 1 (2,3 etc.)’; this way, the name and other personal details are not on the recording.

### Theme: Role in congregation/church/parish

#### Questions:

Can you tell me a bit more about your congregation and the denomination of which your church/parish is part of?

Which age groups, how many members, cultural and ethnic background, etc.

Can you tell something about your motivation for becoming a reverend/pastor?

What are your daily duties in this community?

What role do you have within your community?

### Theme: Worldview/Faith

#### Questions:

What role does your faith/worldview play in your daily life?

What is most valuable to you about your faith?

What beliefs are important to you?

### Theme: Human Germline Gene Editing

Among other things, the video talks about editing embryo DNA to prevent a serious inherited condition Currently, this technique is neither safe nor permitted. In theory, the DNA of an embryo is edited in the lab at a very early stage, after which the embryo is placed in the mother’s uterus. The child and all subsequent generations will not have the genetic conditions because the predisposition for the disease has been cut out of the DNA, as it were. This procedure always requires IVF.

### Questions:

What do you think about modifying embryo DNA to prevent genetic conditions?

To what extent does your worldview/faith play a role in your ideas about human germline gene editing?

Which (Bible) texts, scriptures or other sources do you use as a starting point or as support for your view?

### Possible follow-through questions:

To what extent do you see positive sides to Human Germline Gene Editing? Which are they?

To what extent do you have doubts about Human Germline Gene Editing? If so, which ones?

What influences your view on this the most?

In your opinion, to what extent should people be allowed to decide for themselves whether they want to use this technique as soon as it is medically safe? Why or why not?

In which cases would you or would you not choose to modify the DNA of an embryo? To what extent does the type of disorder or disease play a role in your opinion?

To what extent do you think we are pushing boundaries when we alter the DNA of embryos? What boundaries are these?

To what extent is there a difference between your opinion and the opinion of your congregation on the subject of altering embryo DNA? If there is a difference, can you explain it?

### Theme: Dealing with embryos

To be able to edit embryo DNA in a medically safe and effective manner in the future, a great deal of research is needed, including research on human embryos. In the Netherlands, it is forbidden to create embryos specifically for research. Only embryos left over after IVF treatment may be used for research, with the parents’ permission. These embryos may be kept in the laboratory for 14 days and are then destroyed. Scientists indicate that they need more embryos and at an earlier stage, namely during the first cell division, to conduct proper research and to make the technique of Human Germline Gene Editing safer and more effective. This requires that embryos are created specifically for this research and are then discarded.

### Questions:

How do you feel about the ‘creation’ of human embryos specifically for research purposes?

In your opinion, when does human life begin?

To what extent do these ideas play a role in your feelings about human germline gene editing?

How do you feel about the beginning of life outside the womb?

Which (Bible) texts or scriptures do you take as a starting point in this regard?

### Possible follow-through questions:

To what extent do you have doubts about research on embryos?

To what extent do you see a difference between using ‘left-over’ embryos and ‘created’ embryos specifically for research?

To what extent do you consider an embryo to be a human being?

To what extent do you see a difference in modifying an embryo and modifying egg- or sperm cells?

### Theme: The ‘role’ of suffering

#### Questions:

What meaning does the theme of ‘suffering’ have in life? How would this change if Human Germline Gene Editing were to be allowed?

To what extent does the theme of suffering, or living with a disability or illness, play a role in your opinion on the use of Human Germline Gene Editing?

To what extent does your idea about this play a role in your opinion on modifying embryo DNA?

To what extent does your faith or worldview play a role in this?

### Theme: (Genetic) identity

European legislation on genetic modification talks about the term ‘genetic identity’ assuming some kind of fixed form of human identity determined by a person’s DNA. There is much debate about this term, which is also not clearly defined. I am curious to hear your views on the subject of identity and DNA.

### Questions:

What do you think of when you hear the term genetic identity? To what extent does genetic identity correspond to just ‘identity'?

### Follow-through questions:

To what extent does genetic modification change the identity of an embryo? What are your first thoughts and ideas about this?

In your opinion, when does a person's identity begin?

To what extent can a person’s identity be influenced or changed?

To what extent can a person’s identity be changed?

To what extent is illness a part of a person’s identity?

### Theme: Pastoral care

#### Questions:

If members of your community come to you in the future for pastoral care or advice on HGGE, what would you advise?

Follow-through questions:

In the following situations?

- Prenatal screening for inherited diseases
- IVF
- Embryo selection
- Modifying the DNA of an embryo

Can you explain?

### Theme: Vision of the future

#### Questions

How will Human Germline Gene Editing affect the future?

### Follow-through questions:

In your view, what in particular will change if we start practicing Human Germline Gene Editing?

To what extent are you concerned that genetic modification would make the world more malleable?

What are you most concerned about in that respect?

What do you see as the biggest benefits of genetic modification?

To what extent do you think everything that is technologically possible is also allowed?

What role does your worldview/faith play in this?

To what extent do you think international legislation regarding genetic modification is important?

What do you think should be included in these laws?

What is most important to you when it comes to genetic modification?

Is there anything that was not covered in the interview that is important to you and that you would like to add?

### Background questions

#### Short questionnaire to be taken at the end of the interview

You are: male/female/other *(please circle what applies).*

What is your age?… years old.

What is your nationality?

What is the highest level of education you have completed? *(Tick one answer).*

- No education (primary education: not completed).
- Primary education (primary school, special primary education).
- Primary or preparatory vocational education (such as LTS, LEAO, LHNO, VMBO-k).
- Secondary general secondary education (such as MAVO, (M)ULO, MBO short,VMBO-t)
- Senior secondary vocational education (such as MBO-long, MTS, MEAO, BOL, BBL, INAS).
- Higher general or preparatory scientific education (such as HAVO, VWO, Athenaeum, Gymnasium, HBS, MMS)
- Higher vocational education (HBO, HTS, HEAO)
- Scientific education (university).
- Other, namely …………………………………………

What is your profession or position within your church or congregation?

Within which denomination do you have an active leadership role?

Do you consider your denomination/church as liberal, conservative or more in between.

Can you explain why you see it that way?

## Self-placement of Denomination on the Liberal-Moderate-Conservative Spectrum

**Table:**

Participant	Self-placement of denomination (Liberal – Moderate – Conservative)	Reasoning
#1	Conservative	“That is to say, not liberal, but rather with the Word at the center […] The DNA of the congregation is quite conservative.”
#2	Conservative	“The familiar, timeless message of Jesus Christ as Savior and Lord. But presented in a contemporary form.”
#3	Conservative	“In many respects, [my church] is a liberal denomination (regarding gender, sexual orientation, lifestyle, social engagement). But when it comes to the beginning of life (abortion and human germline gene editing) and the end of life (euthanasia), most of our people are very reluctant, just as there is great restraint toward other interventions in the natural course of events, such as pesticides, artificial fertilizers, IT and social media for children, synthetic materials, and so on.” “So yes, we might not fit neatly into the straightforward picture of conservative–liberal, it’s all a bit more nuanced. Maybe there’s room in your table for an asterisk or another kind of footnote. Because of course, misrepresenting the truth is not an option in a scientific publication.”
#4	Moderate	“[My denomination] called itself ‘the village church’, and that’s what it was, both literally and figuratively. The church building is located in the middle of the village […], and traditionally, a large part of the village belonged to it. Of course there were differences of opinions, some were very liberal, while others preferred to hold on to what was familiar. But in general, people always managed to meet somewhere in the middle.”
#5	Conservative	"[My denomination] came into being because the orthodox group tolerated the Lutheran Church. That means [my church] is a church of compromise. Especially on (medical-) ethical issues, [my church] is fairly conservative."
#6	Conservative	“Most pastors and denominations, except for the more liberal ones, take a cautious stance toward human germline gene editing. Primarily because it involves intervening in, altering, or manipulating human genetic material — the very fabric of life as created by God. It touches upon the uniqueness of God’s creation So rather conservative."
#7	Moderate	“[My denomination] can be situated between orthodox and liberal; leaning somewhat more toward liberal.”
#8	Conservative	“[My church] wants to be faithful to Christ and the Church, open to whatever new things the Holy Spirit may bring and committed to passing on the richness of the Tradition."
#9	Moderate	“Perhaps somewhere in between. Liberal in terms of pastoral approach, traditional in doctrine. A church service has a high liturgical character and of course there are dogmas, but the approach is predominantly pastoral. Married priests, also women in ordained ministry, openly homosexual priests and other clergy and blessing of, for example, relationships other than between a man and a woman. Everyone who participates in the Lord’s Supper or Communion within their own church tradition is welcome to do so with us as well. There is also cooperation and intercommunion with, for example, Anglicans."
#10	Conservative	“I believe in the supernatural reality of Jesus Christ.”
#11	Liberal	"Undogmatic, liberal. Traditionally open to the world (societal issues)."
#12	Moderate	“[My church] is not uniform and I find myself somewhere in the middle.”
#13	Conservative	"Because the authority of God and of Scripture is not seen as a matter of personal choice, but as a given that imposes itself.”
#14	Liberal	N/A
#15	Moderate	"I live within a rich religious tradition. That tradition needs to move with the times.”
#16	Liberal	“Freedom of thought; no dogmas.”
