# Supplementary material for: Dutch Christian Faith Leaders Deliberating Human Germline Gene Editing: A Qualitative Study
Source: J Relig Health. 2026 Mar 29;65(5):4654–84. doi: 10.1007/s10943-026-02637-y (PMC13717633; doi:10.1007/s10943-026-02637-y)
Supplement: Supplementary file 1 [file 10943_2026_2637_MOESM1_ESM.docx]

**Supplementary Information: Information Letter**

**Deliberations of Christian faith leaders regarding Human Germline Gene Editing**

Dear Sir/Madam,

**Information letter**

In this letter, we inform you about our scientific research entitled: ‘Deliberations of Christian faith leaders regarding Human Germline Gene Editing. This research, which consists of personal interviews, is taking place as part of the project ‘The DNA dialogues’ and is being conducted by a research team at the Amsterdam UMC.

Before you decide whether you want to participate in this study, we would like to explain what the study entails. Participation in the study is voluntary. If you want to participate, your written consent is required. We hope you will want to participate in this study!

**Reason**

Human Germline Gene Editing involves modifying the DNA of an embryo in the laboratory and then placing it in the mother's womb. From there, a baby develops that carries the incurred, irreversible modifications in all the cells of their body. This means that the adaptations are also passed on to subsequent generations. As a result, this technique could potentially prevent serious hereditary conditions, such as Huntington's disease, in new generations. In the Netherlands, it is currently forbidden to alter the DNA of future generations. Research is already being conducted into altering DNA of human germline cells, but no pregnancy may yet result (Embryo law art. 24 sub g).

**Purpose of the study**

The discovery of new DNA techniques has accelerated Human Germline Gene Editing. Because Human Germline Gene Editing has potentially far-reaching consequences, it raises ethical questions about what the use of this technique might mean for individuals but also for our society, now and in the future. Decisions on whether or not to allow Human Germline Gene Editing require careful consideration in which not only the views of scientists or politicians are important but also those of the wider public and, within that, of specific groups, such as patients. We believe it is important to contribute to the public dialogue from different (worldview-based) perspectives. This research is part of that. In this research, through interviews, we specifically want to explore the considerations of Christian faith leaders on the development and application of Human Germline Gene Editing.

**What are "The DNA dialogues"?**

In September 2022, the project ‘De DNA dialogen’ ([www.dednadialogen.nl](http://www.dednadialogen.nl/)) was launched with the aim of identifying the views, needs and values regarding Human Germline Gene Editing in society. This information can be used to create appropriate laws and regulations on whether or not to modify embryo DNA. The 'Nationale Wetenschapsagenda' (NWA) makes this research possible. Besides Amsterdam UMC, the project also involves Erasmus MC, the Rathenau Institute, Fontys Hogeschool, Nemo Kennislink, VU Amsterdam, Willem de Kooning Academy, the Erfocentrum, and various associations representing medical professionals or certain patient groups.

**What does participating mean?**

Participants in this study will be interviewed for about an hour. The interview will be recorded. In the interview, questions will be asked about their considerations, thoughts and opinions regarding the modification of embryo DNA and the role their worldview may play in this. Prior to the interview, participants will be sent a short video with information about this new technology.

**Voluntary participation**

You decide whether to participate in the study. Participation is voluntary. Are you participating in the study? Then you can change your mind at any time. You may stop during the study. You do not have to say why you are stopping. However, you must notify the researcher of this decision.

**Use and retention of your data**

This research requires the collection and use of your personal data.

The interview will be recorded and transcribed, no names will be mentioned in the transcript. You will therefore never be recognizable in any publication. Also, your personal information from the interview will not be traceable to your personal data, as the transcript and your consent statement will be kept in two separate places. Only the researchers involved can link the transcript of your interview to the consent statement and short questionnaire by means of a code.

If you wish, you will receive the interview transcript so that you know what data we will store. After agreement, we can use quotes from that text (transcript) in our reporting. Interview transcripts are kept for 10 years after the last publication. Audio recordings are destroyed immediately after they are transcribed. When you sign the consent form, you consent to the collection, storage and viewing of your personal data and the interview. We keep your consent statement 10 years separate from other research data. After that, the personal data will be destroyed.

**Any questions?**

If you have any questions, please contact Wendy Geuverink, one of the researchers at Amsterdam UMC. Telephone: 0645752530. E-mail: [w.geuverink@amsterdamumc.nl](mailto:w.geuverink@amsterdamumc.nl)

**Do you have any complaints?**

If you have any questions or complaints about your privacy, you can first discuss them with the research team. You can also go to the Data Protection Officer at [privacy@vumc.nl](mailto:privacy@vumc.nl) . Or you can complain to the Personal Data Authority at <https://autoriteitpersoonsgegevens.nl/>.

We would greatly appreciate your participation in this study. If so, you are kindly requested to email [w.geuverink@amsterdamumc.nl](mailto:w.geuverink@amsterdamumc.nl). You will then be contacted, either by phone or email.

Thank you very much for your time and consideration,

Wendy Geuverink (MSc, MA)
